# Optimized air–liquid interface cultivation enhances survival and integrity of mouse colon precision-cut tissue slices

**DOI:** 10.1038/s41598-026-67282-7

**Published:** 2026-09-09

**Authors:** Daniela Thalheim, Jessica Knittel, Katharina Erlenbach-Wuensch, Roland Nagy, Rainer Tietze, Arndt Hartmann, Regine Schneider-Stock

**Affiliations:** 1 Institute of Pathology, Experimental Tumorpathology, Universitätsklinikum Erlangen, Friedrich-Alexander-Universität Erlangen-Nürnberg, Universitätsstrasse 22, 91054 Erlangen, Germany; 2 Institute of Applied Quantum Technologies (AQuT.), Friedrich-Alexander-Universität Erlangen-Nürnberg, Erlangen, Germany; 3 Department of Otorhinolaryngology, Head and Neck Surgery, Section of Experimental Oncology and Nanomedicine (SEON), Universitätsklinikum Erlangen, Friedrich-Alexander-Universität Erlangen-Nürnberg, Erlangen, Germany

**Keywords:** Organotypic tissue culture, Histology, Colon, Oxygen, Extracellular matrix, Biological techniques, Biotechnology, Cell biology, Stem cells

## Abstract

Mouse colon precision-cut tissue slices (cPCTS) serve as valuable models for studying physiology, toxicology, and immunology. They retain a three-dimensional structure with physiological cell composition and intact metabolic and immune functions. Additionally, cPCTS support the 3R principle by reducing animal use. This study aimed to optimize cPCTS cultivation to maintain cell integrity, minimize damage, and enhance survival of the multilayer intestinal structure. We established an air–liquid interface (ALI) cultivation system, where cPCTS rest on a semi-permeable insert membrane with medium beneath and direct air exposure above. We compared the effects of different oxygen concentrations on cPCTS survival using LDH assays, TUNEL staining, qPCR, and immunohistochemistry. cPCTS preserved the characteristic colonic architecture for up to 120 h, with optimal tissue integrity maintained for at least 96 h. Compared with 80% O_2_, cultivation at atmospheric O_2_ significantly reduced oxidative stress and DNA damage, as demonstrated by lower expression of oxidative stress-associated genes and fewer γH2AX-positive cells. Reduced apoptosis and cytotoxicity were confirmed by less TUNEL-positive cells and lower LDH release. Lower oxygen further preserved crypt morphology, increased *Lgr5* and *Mki67* expression, and resulted in higher numbers of Ki67-positive cells, indicating improved stemness and proliferation. Histological analyses additionally revealed a more physiological extracellular matrix remodeling response under atmospheric O_2_. ALI cultivation simplifies medium changes and facilitates experimental manipulation of the system. Lower oxygen conditions improve long-term survival and structural integrity of cPCTS providing a robust platform for extended mechanistic and co-culture studies.

**Supplementary Information:**

The online version contains supplementary material available at https://doi.org/10.1038/s41598-026-67282-7.

## Introduction

In recent years, the 3R principles (Replacement, Reduction, and Refinement) have become increasingly important for the establishment and optimization of research models across various biological contexts^1^. Colon precision cut tissue slices (cPCTS) present a promising alternative that incorporates both the principles of reduction and refinement. These slices can be utilized in a variety of applications, including drug transport studies^2^, or as a model for food allergic reactions^3^ and microbial interactions^4^. CPCTS retain the natural, three-dimensional cellular composition of the individual intestinal layers, preserving their metabolic and immunological functions.

One significant advantage of cPCTS is the high number of slices that can be generated from a single organ, allowing for the investigation of a wide range of experimental conditions using the same tissue origin. Laboratory animals are particularly advantageous in this context due to their ready availability and genetic consistency compared to human samples^5^. Furthermore, complete cross-sections of the murine colon can be cultivated, which means that every cell type of the colon wall is present. This is not possible with human samples due to the size/thickness of the human intestine.

Since very different cell types of the tissue are present in a multilayer structure, it is demanding to find optimal culture conditions. The primary challenge is to maintain cell integrity as much as possible over a longer period, or even to promote regeneration of cells by the tissue. One of the main technical problems is the early regression of the crypts, with significant decreases observed after just 24 h due to the physical damage by slicing. Additionally, both stem cells and proliferating cells die and cannot be renewed by the slices. To address this, studies have shown that the lifespan of cPCTS can be extended by adding growth factors, which are also used for cultivating intestinal organoids^5^. However, supplementation with growth factors is associated with high costs, especially for experiments conducted over extended time intervals.

One possible reason for the reduced viability of cPCTS is an insufficient supply of oxygen to the tissue, as oxygen dissolves poorly in fluids. Koch et al. demonstrated that the addition of perfluorodecalin (PFD), an oxygen transporter, improved oxygen solubility in liver precision cut tissue slices, leading to extended lifespan. However, the interaction between PFD and tissue remains unclear and could potentially influence examination results^6^. Another possibility is air–liquid cultivation, primarily used for lung precision cut tissue slices. In this technique, the slices are placed on inserts with a semi-permeable bottom. The medium is placed beneath the slices, allowing them to have direct contact with the ambient air from the incubator, thereby providing the necessary oxygen^7,8^.

The aim of this work was to optimize the cultivation system for cPCTS to maintain health and cell integrity throughout the cultivation period, to minimize cellular damage and to increase the survival of the multilayer intestinal structure.

## Materials and methods

**Fig. 1:**
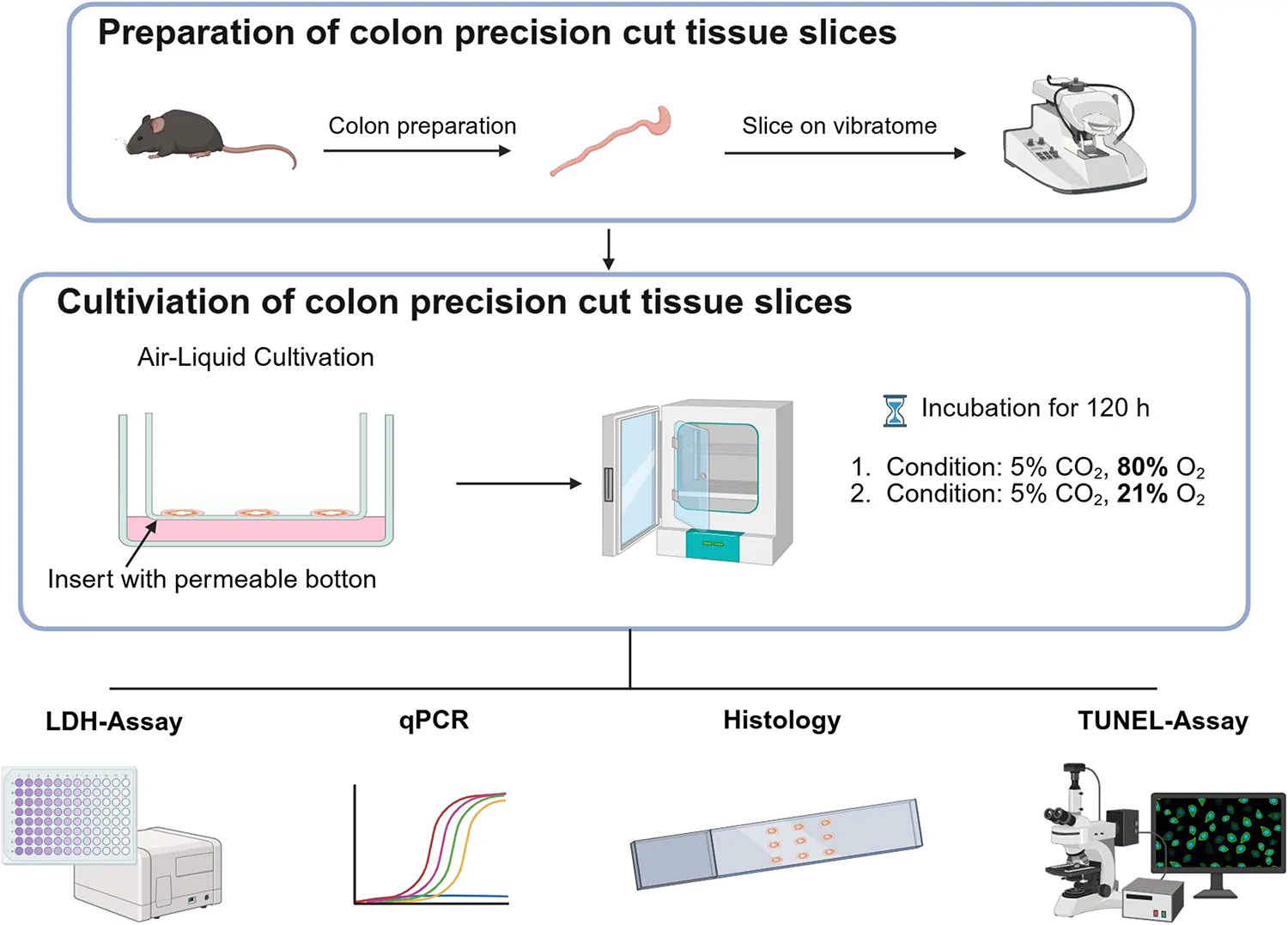
Schematic illustration of the experimental setup. Precision-cut tissue slices (PCTS) were generated from mouse colon using a vibratome. The colon PCTS (cPCTS) were cultured for 120 h using the air–liquid interface (ALI) technique. To assess tissue integrity and viability over time, LDH assays, qPCR, histological staining, and TUNEL assays were performed.

The methodical workflow is shown in Fig. 1. The preparation and cultivation of cPCTS was developed in our lab in 2022^9^ and now further adapted and improved.

### Mouse experiments

For the preparation of cPCTS, colons were obtained from female C57BL/6 mice (The Jackson Laboratory, Bar Harbor, ME, USA), aged six to eight weeks and weighing 16–20 g. The mice were maintained until experimental use in the preclinical animal facility “Franz-Pezoldt-Zentrum” (FPZ). Animals were housed and bred in individually ventilated cages under a 12 h light/12 h dark cycle, with ad libitum access to food and water.

All procedures involving mice, including breeding, housing, and experimental use, were conducted in accordance with the guidelines of the local Institutional Animal Care and Use Committee (IACUC) of Friedrich-Alexander-Universität Erlangen–Nürnberg and the regulations of the Government of Lower Bavaria and all methods were approved by the local Institutional Animal Care and Use Committee (IACUC) of Friedrich-Alexander-Universität Erlangen–Nürnberg (reference number TS-16/22). Animal euthanasia and organ collection were approved in accordance with § 4 (3) of the German Animal Welfare Act (TierSchG) and the ARRIVE 2.0 guidelines.

### Preparation of cPCTS

After death was confirmed by the absence of respiration and heartbeat, the colon was immediately prepared, and directly flushed with ice-cold oxygenated Krebs–Henseleit buffer (KHB). The colons were filled with 3% low melting agarose (Sigma-Aldrich Chemie GmbH, Taufkirchen, Germany). 200 µm thick PCTS were prepared with a Leica VT 1200S Vibratome (Leica Mikrosysteme Vertrieb GmbH, Wetzlar, Germany) in cold KHB (Suppl. Table 1).

### Cultivation of cPCTS

For air–liquid (ALI) Cultivation, cPCTS were incubated in ThinCert™ Tissue Culture Inserts (Greiner Bio-One GmbH, Frickenhausen, Germany) in 6-well plates with three cPCTS in each well. Inserts with a pore size of 0.4 µm and a pore density of 6.1 × 10^8^ pores/cm^2^ were used. The medium was given in the lower chamber. They were cultivated in 1.6 mL Advanced Dulbecco’s Modified Eagle’s Medium/Nutrient Mixture F12 (Gibco™, Life Technologies, Bleiswijk, The Netherlands) supplemented with 25 mM d-Glucose, 2 mM GlutaMAX (Gibco™, Life Technologies, Bleiswijk, The Netherlands), 1% Penicillin/Streptomycin (PAN-Biotech, Aidenbach, Germany) and 100 µg/mL Primocin (InvivoGen Europe, Toulouse, France) at 37 °C, 5% CO_2_ and atmospheric air or 80% O_2_. Medium was changed every 24 h.

### Viability

The viability of the slices was detected by a lactate dehydrogenase (LDH) Assay (Roche Diagnostics GmbH, Mannheim, Germany) in 96-well plates after 24 h, 48 h, 72 h and 96 h as previously described by Neuhaus et al.^10^. Three cPCTS were cultured for 24 h in PBS as a death control and LDH levels in experimental conditions were expressed relative to this control. The measurement was performed with a PerkinElmer 2030 Multilabel reader (PerkinElmer, Inc., Waltham, USA) in duplicates.

Furthermore, a terminal deoxynucleotidyl transferase dUTP nick end labeling (TUNEL) assay (In Situ Cell Death Detection Kit, Sigma-Aldrich Chemie GmbH, Taufkirchen, Germany) was performed according to the manufacturer’s protocol to show the vitality. Slices were imaged using a LSM710 microscope (Zeiss AG, Oberkochen, Germany).

### QPCR

For qPCR nine slices were pooled after different incubation time points (0 h, 24 h, 48 h, 72 h, 96 h, and 120 h), snap-frozen, stored at – 80 °C until RNA isolation. RNA isolation was made with QIAzol Lysis Reagent (Qiagen GmbH, Hilden, Germany) and a clean up of the isolated RNA was made with the RNeasy Mini Kit (Qiagen GmbH, Hilden, Germany) according to the manufacturer’s protocol. RNA quantity was determined by using a NanoDrop™ ND-1000 (Thermo Fisher Scientific, Erlangen, Germany). Reverse transcription was performed with the QuantiTect® Reverse Transcription Kit (Qiagen GmbH, Hilden, Germany) with an input of 500 ng RNA. QPCR primer sequences are given in Suppl. Table 2. The QuantiTect SYBR® Green PCR Kit (Qiagen GmbH, Hilden, Germany) was used and qPCRs were run on a CFX96 Touch Real-Time PCR Detection System (Bio-Rad Laboratories, California, USA). MRNA gene expression was normalized against the housekeeping gene Eukaryotic Elongation Factor 2 (*Eef2*).

### Immunohistochemistry

CPCTS were fixed in 4% buffered formalin (AppliChem GmbH, Darmstadt, Germany) for 30 min and processed for immunohistochemistry (IHC) analysis. Paraffin-embedded tissue was cut into 1 µm thick sections. IHC staining for haematoxylin and eosin, PAS, Ki67, Villin and γH2AX (Suppl. Table 3) was performed and stained sections were scanned as described at Huebner et al.^11^. Collagen content and tissue fibrosis were assessed by Van Gieson staining of paraffin-embedded tissue sections following standard histological procedures. Ki67 quantitative analyses were performed with the open-source software QuPath 0.5.0 (https://qupath.github.io). Three slices were analyzed for each time point per condition per replicate (3 mice with three replicates, n = 9).

For automated quantification, the built-in “Positive Cell Detection” tool in QuPath was used to determine the percentage of positively stained cells.

### Statistical analysis

Statistical analysis was performed with the python 3 package stat annotations 0.6.0 using the Welch t-test. Statistical significance was defined as *p* < 0.05.

## Results

### Long-term Preservation of all intestinal cell subtypes

**Fig. 2:**
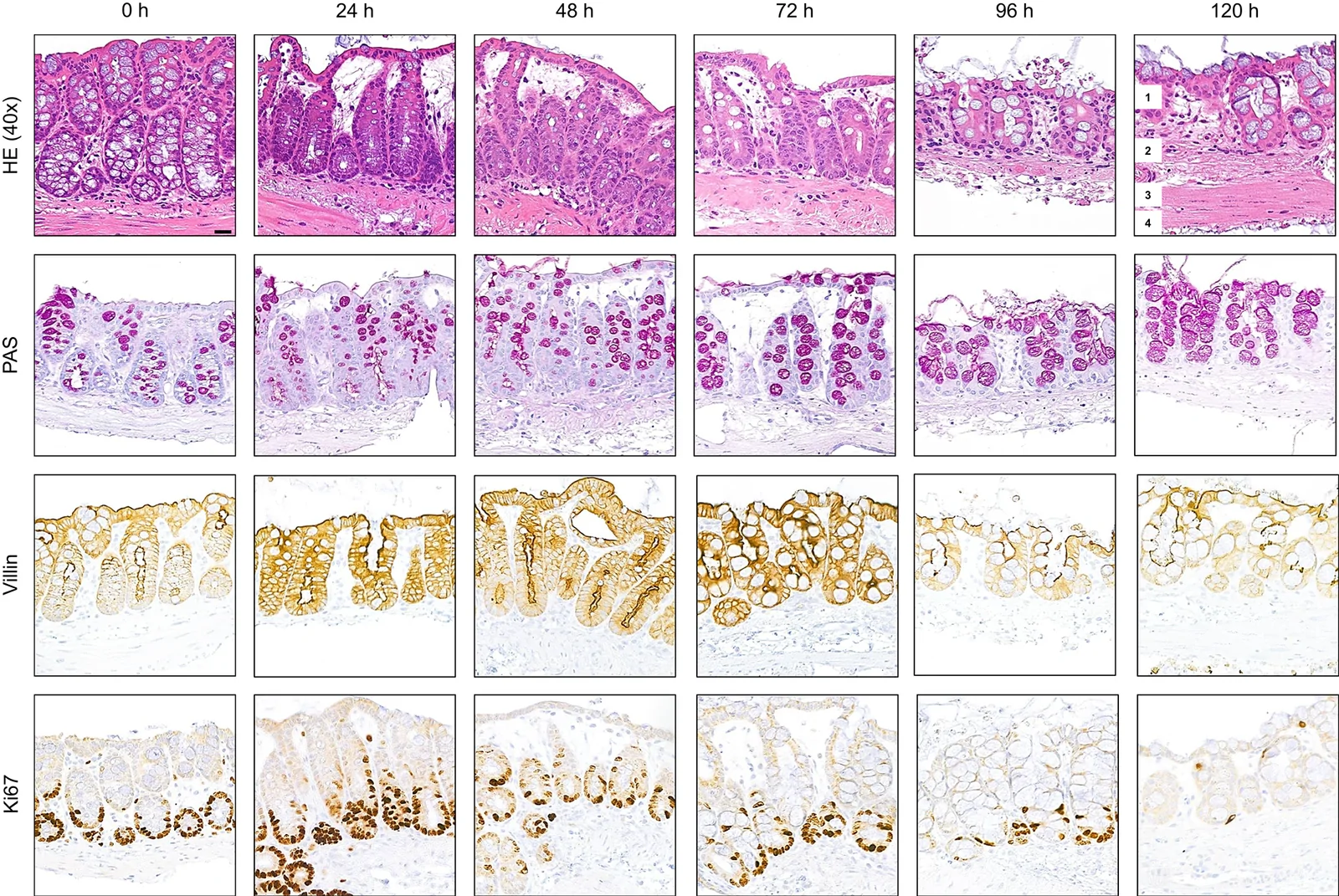
Representative histological sections of cPCTS over time. CPCTS were cultivated with 80% oxygen. H&E staining demonstrated preservation of the intestinal tissue architecture, including the mucosal compartment with intact crypt structures (1), the submucosa (2), muscularis propria (3), and serosa (4). PAS staining highlights goblet cell enlargement, while Ki67 staining shows a progressive decline in proliferating cells. Staining for Villin, a marker for enterocytes, was declining over time. (scale: 20 µm).

We cultivated cPCTS in an air–liquid interface (ALI) condition with 80% oxygen for 120 h. This oxygen level was initially chosen based on its established use in previous studies involving intestinal precision-cut tissue slice models^5,12^. To investigate the vitality of the cPCTS, slices were immunohistochemically stained and evaluated (Fig. 2, Suppl. Fig. 1).

The 0 h time point represents the status of the cPCTS immediately after the cutting process, without any further cultivation. At this initial time point, the tissue most closely resembled native colonic mucosa, displaying a well-preserved crypt architecture with regularly arranged crypts, an intact surface epithelium, abundant PAS-positive goblet cells, continuous apical Villin expression, and Ki67-positive cells predominantly localized to the basal crypt compartment. Villin staining remained detectable throughout the cultivation period, indicating preservation of the epithelial compartment and brush border structures. Villin expression appeared more pronounced at 24 h compared with 0 h before gradually declining at later time points (Fig. 2). This increase coincided with peak Ki67 expression and reduced goblet cell marker expression, suggesting a transient shift in epithelial cell composition during the early phase of cultivation. With prolonged cultivation, progressive structural and cellular alterations became more evident visible as mild edema formation between crypts. A gradual loss and shortening of crypt structures was observed, with partially absent crypts at the 120 h time point. PAS staining showed progressive enlargement and increased mucus accumulation in goblet cells. While Villin expression remained detectable until 72 h, it decreased at 96 h and 120 h and its distribution became less uniform. Ki67 staining demonstrated a continuous decline in proliferative activity, with markedly reduced numbers of proliferating cells at later time points (96 -120 h). Prolonged cultivation was associated with increased signs of necrosis and inflammation, accompanied by reduced cellular integrity. Overall, these findings indicate that epithelial structure and differentiation were largely preserved during the first 48 h of ex vivo culture, whereas extended cultivation resulted in progressive tissue degeneration.

Next, we performed an LDH-assay at different time points to assess cytotoxicity (Suppl. Fig. 2a). Dying or damaged cells release LDH into the surrounding medium. Over time, a gradual increase in LDH release was observed, indicating progressive cell damage. Significantly elevated LDH levels were detected at 72 h, 96 h, and 120 h compared to 24 h. Notably, LDH release at all time points remained below the level of the death control, indicating that complete tissue disintegration was not reached during the cultivation period.

The tissue histology was further confirmed by qPCR (Suppl. Fig. 2b). The markers for enterocytes (*Alpi*, *Cdh1*, *Msn*, *Vil1*) and enteroendocrine cells (*Chga*, *Cldn15*) were expressed at the same or higher levels throughout the entire time course compared to the 0 h time point. This suggests that the cPCTS show differentiation towards these cell types under ex vivo conditions.

The gene expression of *Muc2* was significantly increased at 24 h but decreased over time. Conversely, the expression level of *Atoh1* was significantly reduced after 24 h and continued to decline, indicating that further differentiation into goblet cells did not occur (Suppl. Fig. 2b).

The expression levels of fibrosis markers *Tgfb1*, *Serpinh1*, *Mmp3,* and *Fn1* and of inflammation markers *Il6*, *Tnfa*, and *Il1b* significantly increased over time compared to the status immediately after cutting. (Suppl. Fig. 2b).

The gene expression of stem cell marker *Lgr5* decreased during incubation indicating that proliferating crypt stem cells were not preserved. The proliferation marker *Mki67* was downregulated in qPCR analyses. Similarly, in the Ki67 staining, proliferating cells were abundantly visible at the base of the crypts up to the 72 h time point, though a decline over time was evident. After 96 h a lower number of proliferating cells was visible (Fig. 2, Suppl. Fig. 2c).

In summary, we suggest that cPCTS cultivation was possible for up to 120 h. After 96 h, crypt integrity was decreased along with stem cells and proliferating cells.

### Lower oxygen content in cultivation medium reduces damage by oxidative stress and increases survival

In the ALI configuration, direct exposure to the gas phase circumvents the limitations of oxygen solubility in liquid media. However, this also results in exposure to supraphysiological oxygen levels (e.g., 80%), which may promote oxidative stress and tissue damage. Reducing oxygen levels to atmospheric oxygen therefore serves to lower exposure from hyperoxic to atmospheric conditions, thereby limiting oxidative stress.

**Fig. 3:**
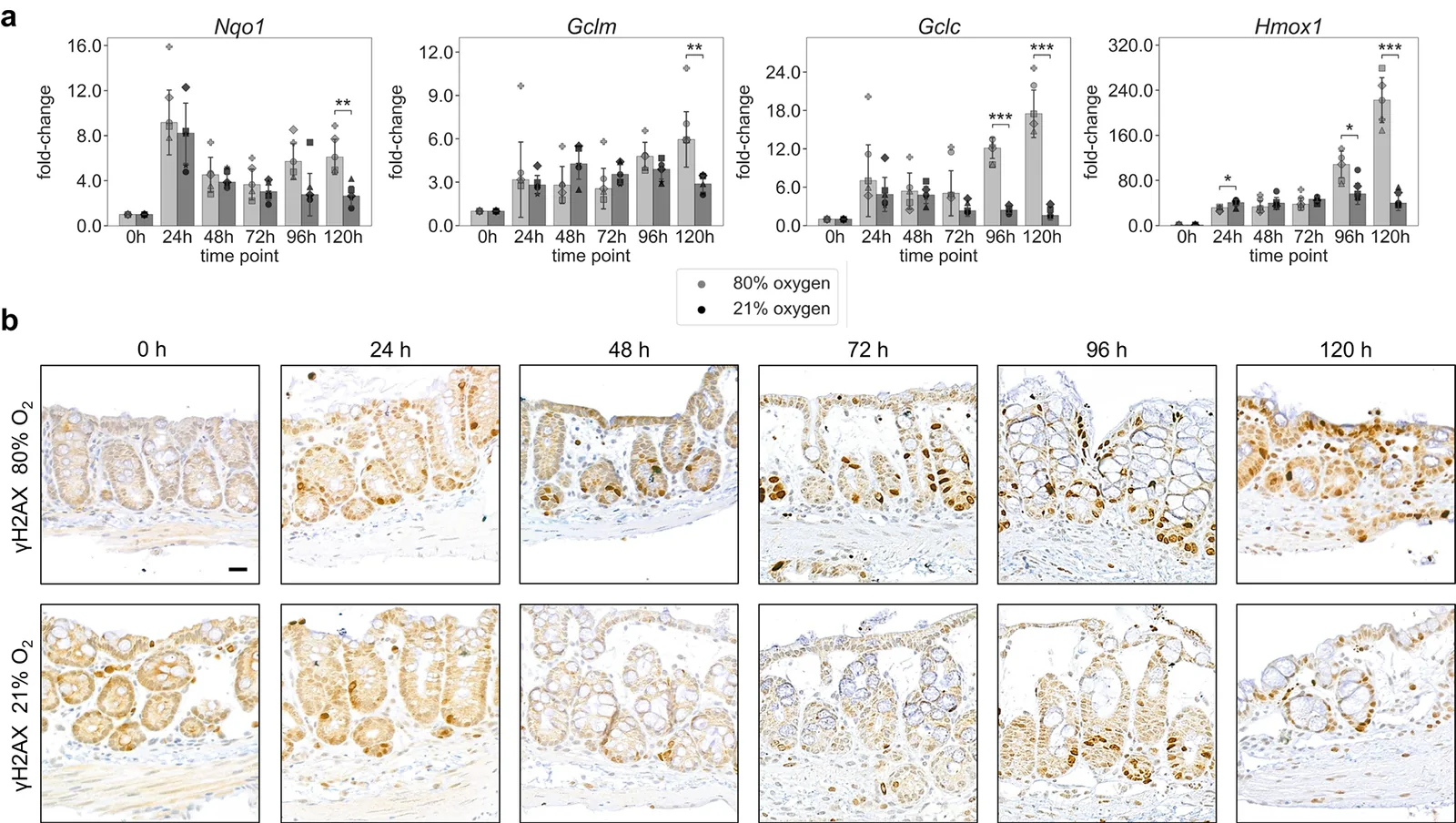
Reduced oxygen stress and apoptosis upon lower oxygen content. (**a**) The gene expression fold changes of different marker genes for oxidative stress were reduced (n = 5) and (**b**) less DNA damage was seen in γH2AX staining at the lower oxygen condition (scale: 20 µm). Welch t-test was used to calculate statistical differences (*p < 0.05, **p < 0.01, ***p < 0.001).

To confirm the reduced oxidative stress response, we tested *Nqo1*, *Gclm*, *Gclc*, and *Hmox1* as markers for inflammation via qPCR (Fig. 3a). Indeed, atmospheric oxygen does not primarily induce an oxidative stress phenotype as reflected by lower *Nqo1* and *Hmox1* expression, unchanged *Gclm* levels, and reduced *Gclc* expression at 72 h. In contrast, 80% oxygen strongly induces oxidative stress-associated genes, particularly *Gclc* and *Hmox1* at 72 h, indicating a pronounced adaptive antioxidant response under hyperoxic conditions.

The reduced oxidative stress was also visible in immunostaining for γH2AX. Although DNA damage was detectable at both oxygen concentrations, there was significantly less DNA damage in the lower oxygen condition compared to 80% oxygen culture (Fig. 3b). We suggest that damage at 0 h reflected the physical stress by the cutting procedure. However, it was reduced after 24 h, which indicates active repair processes and a recovery of the slice vitality. There was no further damage up to 72 h. Then, the number of γH2AX positive cells increased continuously. Notably, in areas with a higher number of γH2AX positive cells, such as in the crypt floor, proliferating cells were also present. In combination with the qPCR results, we suggest that atmospheric oxygen culture reduced the cell damage and improved the vitality of cPCTS.

**Fig. 4:**
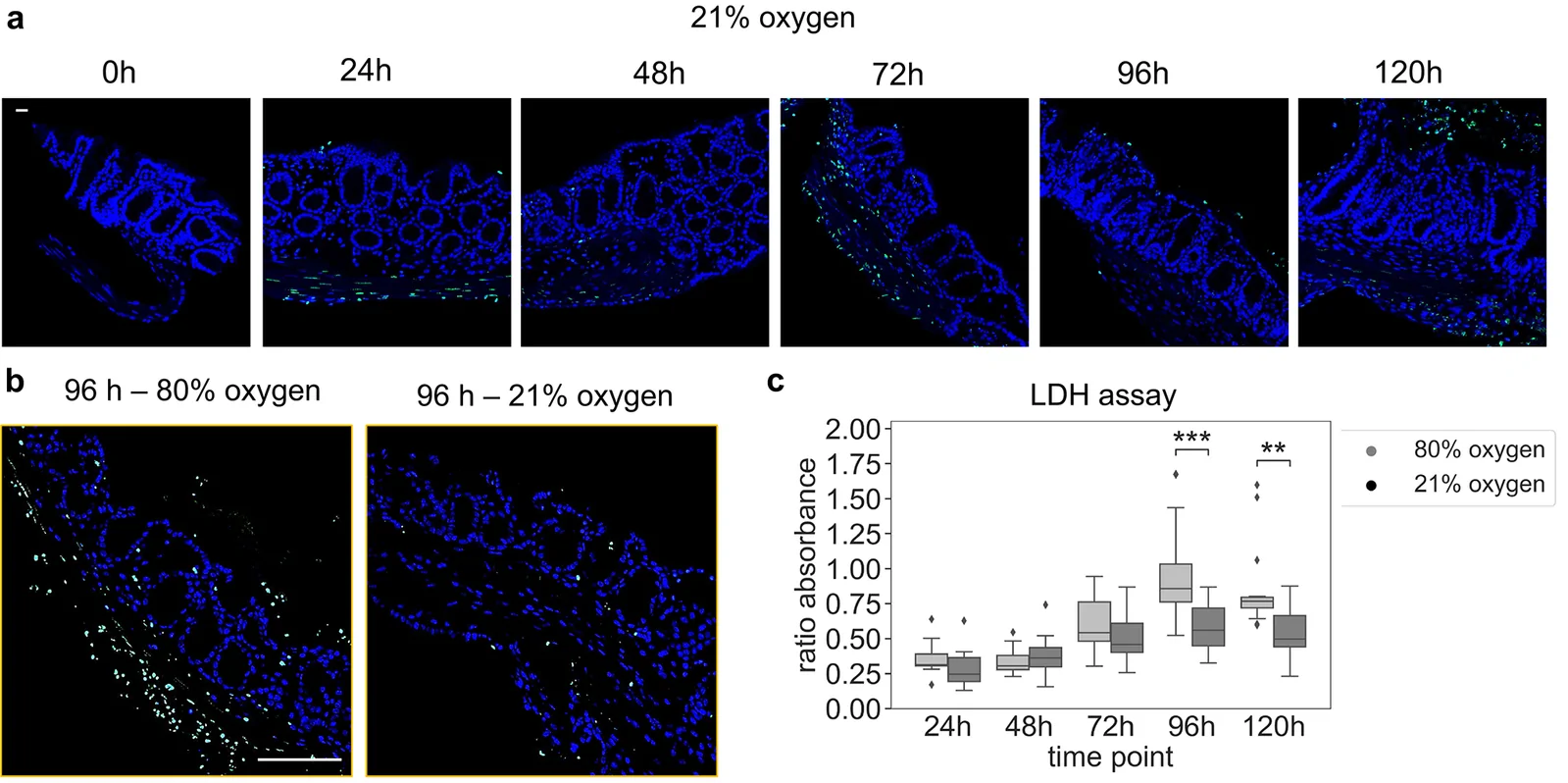
(**a**) TUNEL staining (green) revealed only a minimal increase in cell death over the 96 h culture period under atmospheric oxygen. Nuclei were counterstained with DAPI (blue). Scale: 20 µm. (**b**) Comparison of TUNEL staining (green) between cPCTS cultures maintained at 80% and atmospheric oxygen demonstrated a markedly increased level of cell death under hyperoxic conditions after 96 h. Nuclei were counterstained with DAPI (blue). Scale: 100 µm. (**c**) cPCTS cultured under atmospheric oxygen exhibited significantly reduced LDH release compared with tissues exposed to hyperoxic conditions (6 mice, triplicate cultures; *n* = 18 samples). Welch t-test was used to calculate statistical differences (**p < 0.01, ***p < 0.001).

Next, we examined the overall survival of the tissue. Cell death was first assessed by TUNEL staining. Under atmospheric oxygen, only a slight increase in TUNEL-positive cells was observed over the 96 h culture period (Fig. 4a). Comparison of cultures maintained at atmospheric and 80% oxygen revealed markedly higher levels of cell death under hyperoxic conditions after 96 h (Fig. 4b), whereas no differences in TUNEL-positive cells were observed at 0 h (Suppl. Fig. 3). Positive and negative staining controls are given in Suppl. Fig. 3. In agreement with these findings, cPCTS grown in atmospheric oxygen displayed significantly lower LDH release into the culture medium at 96 h and 120 h, indicating less cytotoxicity compared with those cultured in 80% oxygen (Fig. 4c).

### A reduced oxygen level in the cultivation medium better preserves cell integrity, maintains stemness, and induces a more protective inflammatory response

**Fig. 5:**
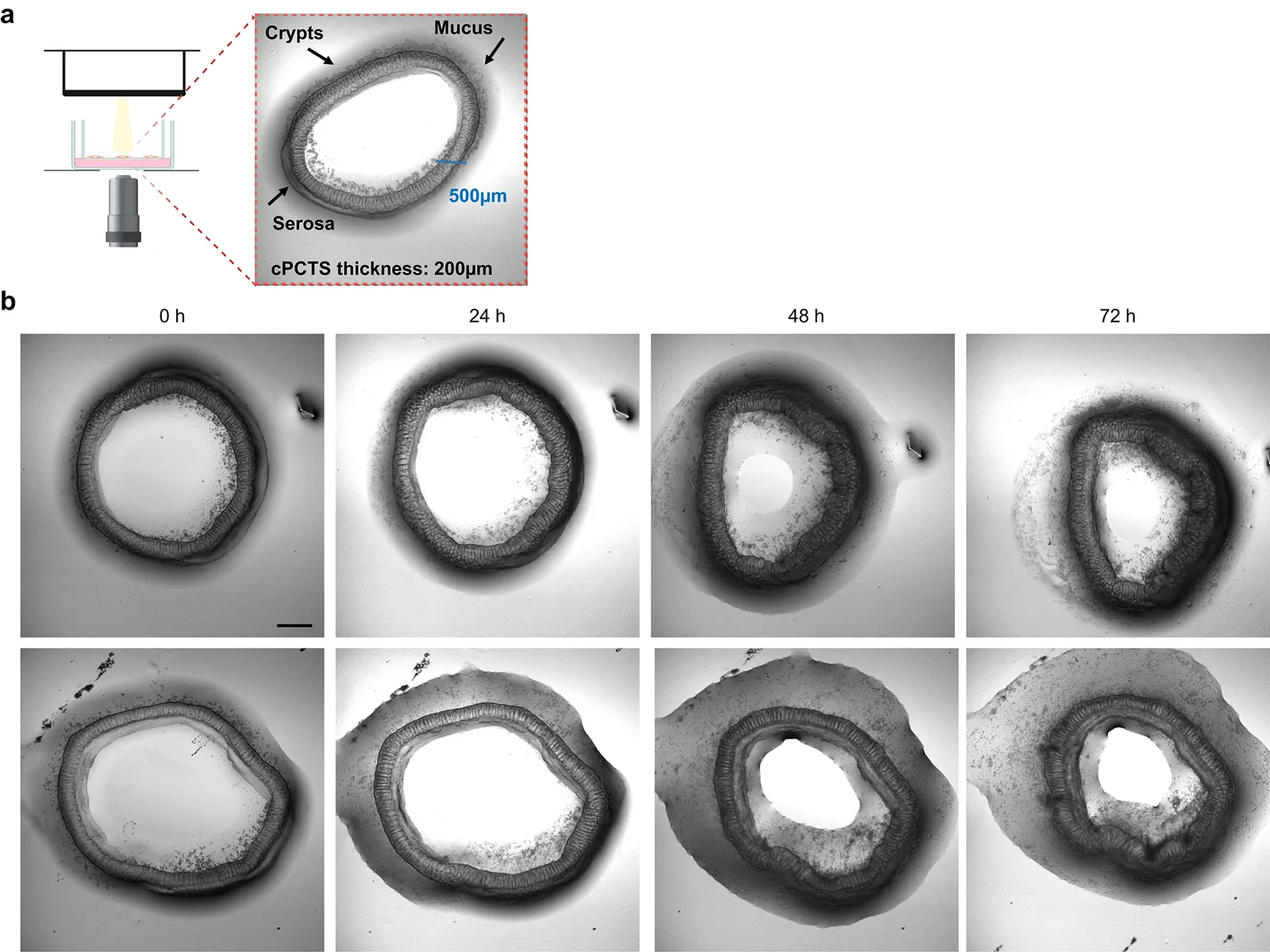
Advantages of ALI cultivation. (**a**) Schematic representation of the microscopy setup used for imaging cPCTS under ALI cultivation conditions and (**b**) representative microscopy images of cPCTS over a 72-h cultivation period. The crypt structures and mucus production are clearly visible. Additionally, the movement of the cPCTS, such as contraction, can be observed over time. Scale: 500 µm (Microscope: Eclipse Ti2, Nikon, Amstelveen, The Netherlands).

ALI cultivation enabled repeated live imaging of the same cPCTS over a 72 h period (Fig. 5a and b). Throughout cultivation, the characteristic crypt-containing mucosal layer remained clearly visible, and mucus production could be observed within the luminal compartment (Fig. 5b). Furthermore, dynamic tissue movements, including spontaneous contractions, were detectable over time. These observations demonstrate that ALI cultivation preserves tissue morphology and functionality while facilitating longitudinal microscopic monitoring.

**Fig. 6:**
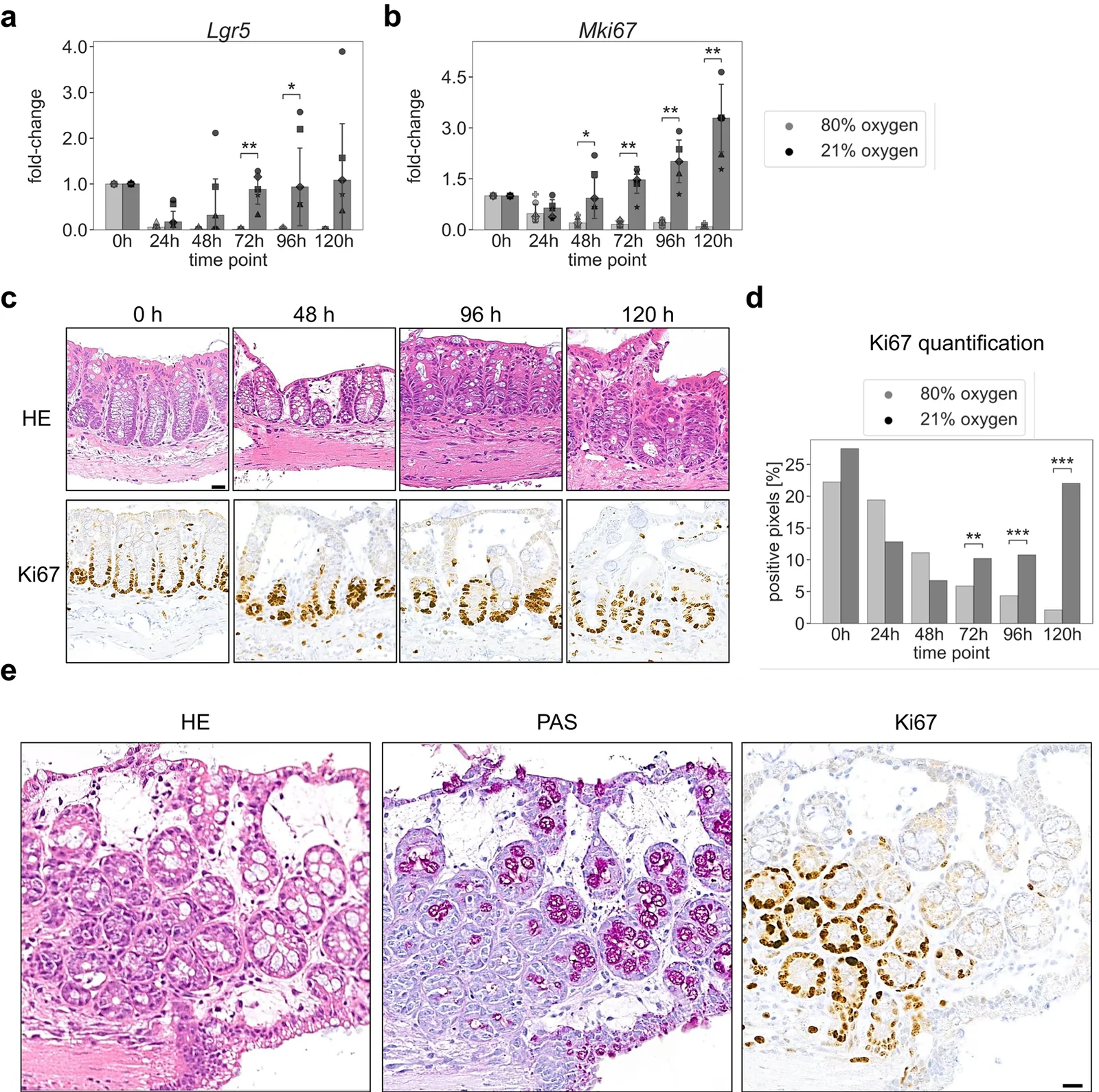
The reduction of oxygen enhances the survival of cPCTS. (**a and b**) The gene expression fold changes of *Lgr5* and *Mki67* were significantly higher, when cPCTS were cultivated at atmospheric oxygen compared to 80% oxygen (n = 5). For 80% oxygen the same dataset was used as in Suppl. Figure 2 b and c. (**c**) Representative Ki67 staining of cPCTS cultivated at atmospheric O_2_ throughout the cultivation period, demonstrating preservation of proliferating cells (scale: 20 µm). (**d**) Quantification of the positive Ki67 cells in histological stainings was significantly higher in atmospheric oxygen-cPCTS compared to 80%-cPCTS (3 mice in triplicate, n = 9 samples). (**e**) Representative histological analysis of cPCTS cultivated at 80% O_2_ (48 h) showing a mutually exclusive pattern between proliferating cells at the crypt base and differentiated goblet cells (scale: 20 µm). Welch t-test was used to calculate statistical differences (*p < 0.05, **p < 0.01, ***p < 0.001).

Since preservation of stem cell population is important for the survival of cPCTS, we performed a qPCR for the *Lgr5* stemness marker. *Lgr5* expression was found to be significantly higher in atmospheric oxygen-cPCTS than in 80%-cPCTS cultures. Although initially downregulated, *Lgr5* levels recovered and even increased over time (Fig. 6a). Proliferating cells were clearly visible, with *Mki67* expression rising steadily, reaching a threefold increase at 120 h compared to 0 h (Fig. 6b). Histological Ki67 staining confirmed these findings, showing more positive cells at atmospheric oxygen than at 80% by the end of cultivation (Figs. 2 and 6c and d). As expected, proliferating cells and differentiated goblet cells remained mutually exclusive (Fig. 6e).

Further histological H&E-analysis showed an improved tissue integrity at atmospheric O_2_, with better preserved crypt structures over time and only minimal oedema and mild inflammation. Villin staining consistently demonstrated the presence of enterocytes throughout the entire time course, and goblet cells showed a tendency to increase in size until 72 h with further decrease at later time points (Suppl. Fig. 4).

**Fig. 7:**
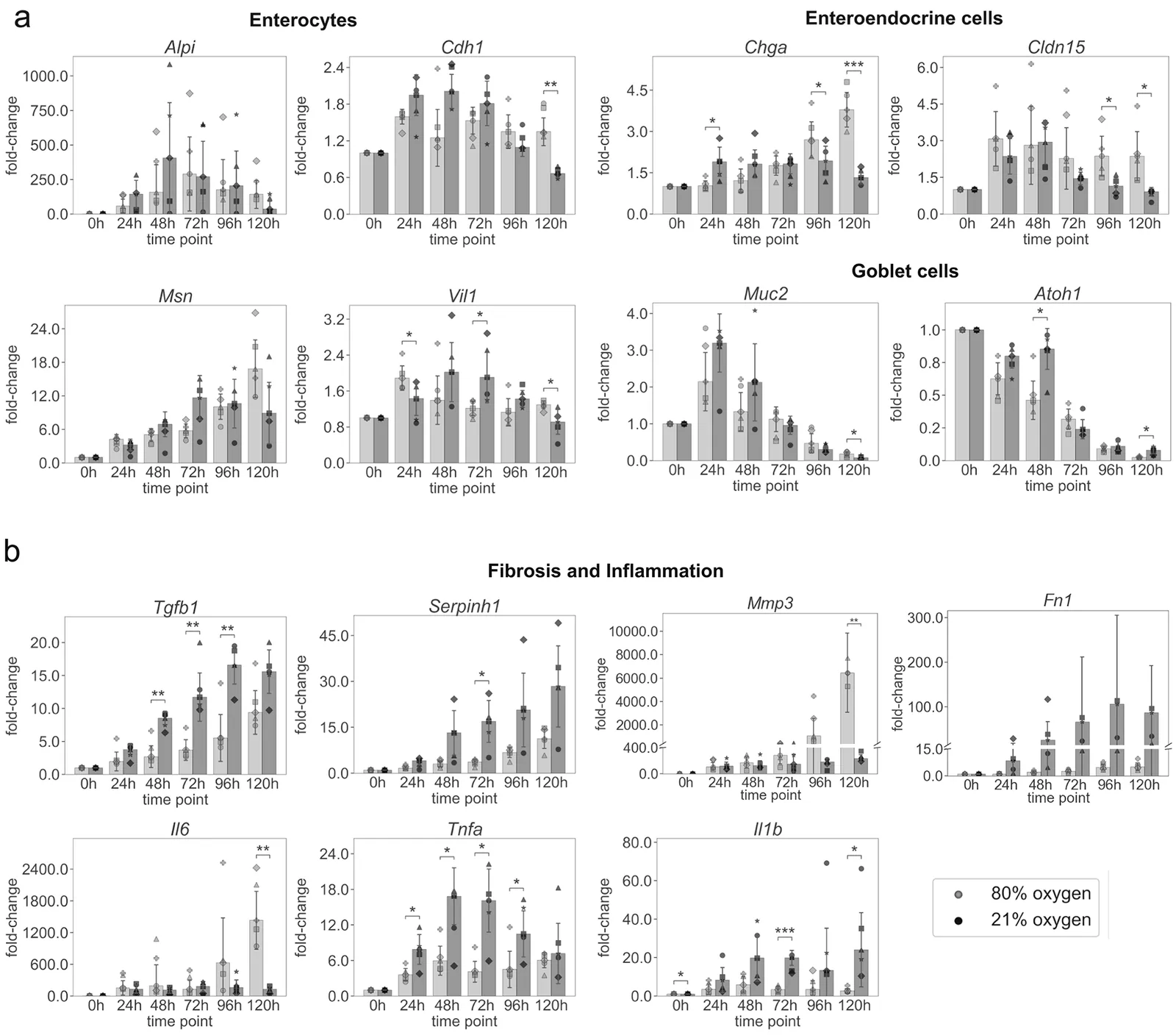
Comparison of the qPCR results for different marker genes in the two different cultivation conditions with 80% and atmospheric oxygen. Welch t-test was used to calculate statistical differences. (n = 5, *p < 0.05, **p < 0.01, ***p < 0.001); For 80% oxygen the same dataset was used as in Suppl. Figure 2b and c.

The histological findings were supported by our molecular analysis of differentiation markers. QPCR analysis revealed that the expression of enterocyte markers (*Alpi, Cdh1, Msn, Vil1*) was elevated at most of the time points, while the expression of enteroendocrine markers (*Chga, Cldn15*) remained rather stable in atmospheric oxygen. Goblet cells were particularly active at 24 h and 48 h, with increased expression of *Muc2* and *Atoh1*, although no significant differences were observed between the 80% and atmospheric oxygen levels at later time points. (Fig. 7a).

When comparing inflammatory and fibrotic markers by qPCR, we observed higher expression levels of *Tgfb1*, *Serpinh1*, and *Il1b*, mostly during the first 72 h. *Fn1* was upregulated, but due to the high variation of data statistically not significant. These data indicated an improved maintenance of immune homeostasis and enhanced intestinal mucosal healing under atmospheric oxygen conditions. As expected, expression of the pro-inflammatory cytokine *Il6* was reduced when the oxygen concentration in the medium was lowered. Additionally, *Mmp3* expression was decreased under atmospheric oxygen, which may reflect better physiological conditions. The increased expression of fibrotic markers under atmospheric oxygen suggests the presence of pathophysiologically active repair mechanisms in the cPCTS (Fig. 7b).

**Fig. 8:**
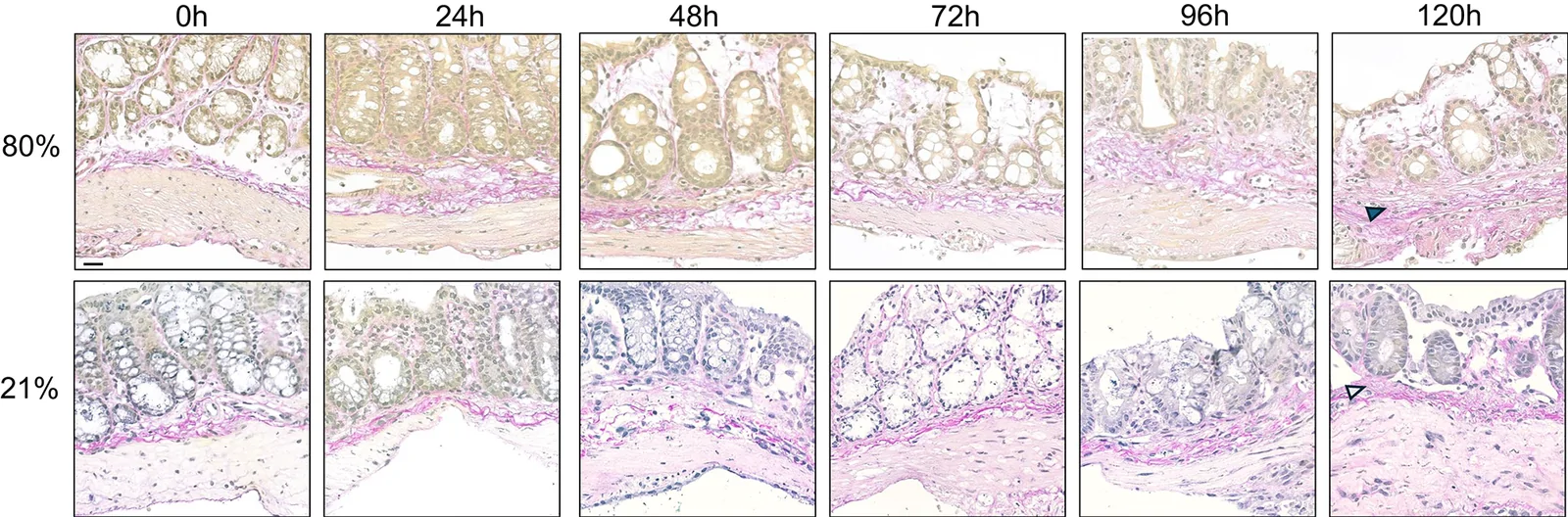
Representative Van Gieson staining of cPCTS cultivated under atmospheric and 80% O_2_ throughout the cultivation period, demonstrating maintenance of the submucosal collagen-containing compartment. Arrowheads indicate representative collagen-positive regions. Scale: 20 µm

**Fig. 9:**
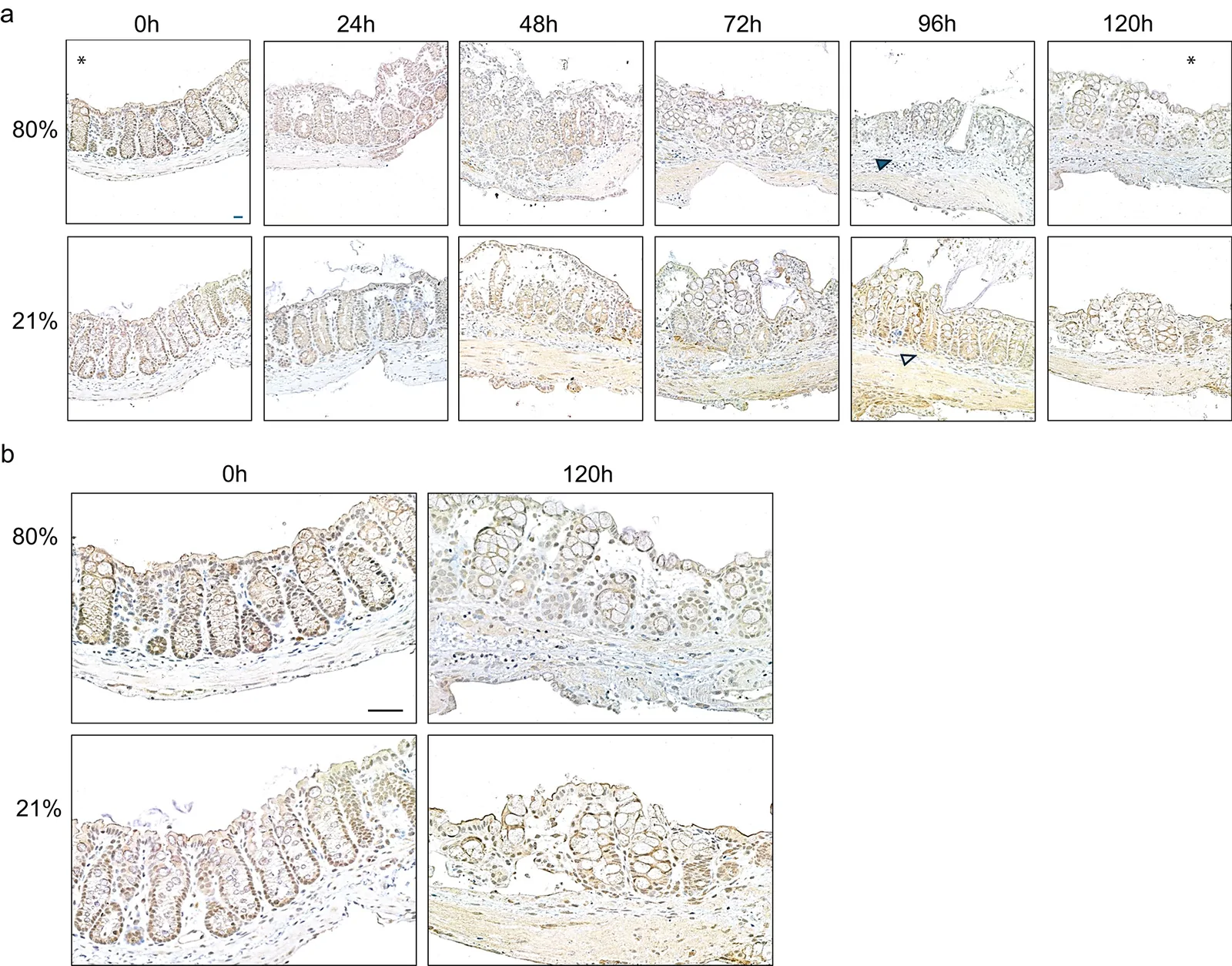
(**a**) Representative Mmp9 staining of cPCTS cultivated under atmospheric and 80% O_2_ throughout the cultivation period. Mmp9 expression was predominantly localized to the epithelial compartment. (**b**) Higher-magnification images of (**a**) (*) illustrate the slight decrease in Mmp9 expression upon 80% O_2_ at 120 h. Arrowheads show the thickness of the collagen-positive submucosal layer. Scale: 20 µm.

Histomorphological assessment of both culture conditions supported the presence of oxygen-dependent matrix remodeling where collagen was primarily localized within the subepithelial stromal tissue underlying the mucosal crypt compartment (Fig. 8). While *Tgfb1* and *Serpine1* expression was elevated in cPCTS cultured under atmospheric oxygen, this was not accompanied by an obvious increase in histologically detectable collagen deposition. Instead, qualitative differences in matrix organization were observed. Under atmospheric oxygen, collagen fibers appeared more compact and organized within a narrower submucosal layer, whereas under 80% oxygen the collagen-positive area was broader and displayed a more loose and disorganized appearance. In parallel, Mmp-9 immunoreactivity predominantly detected in epithelial glandular structures tended to decline over time at 80% O2 (Fig. 9). This trend is consistent with the significantly lower *Mmp3* mRNA expression detected by qPCR (Fig. 7b).

In summary, lowering oxygen levels significantly preserved morphological features, enhanced survival, proliferation, and stem cell marker expression. This indicates that lower oxygen concentrations are particularly beneficial for the long-term cultivation of cPCTS.

## Discussion

Compared with conventional in vitro culture systems, cPCTS offer the distinct advantage of preserving the native tissue architecture and cellular complexity of the original tissue. In addition to maintaining epithelial, stromal, and immune cell populations, the model retains physiologically relevant cell–cell and cell–matrix interactions as well as key features of the tumor microenvironment. These characteristics are difficult to reproduce in traditional two-dimensional cultures and make cPCTS a valuable ex vivo platform for mechanistic studies and the investigation of tumor-microenvironment interactions under conditions that more closely resemble the in vivo situation. However, maintaining tissue integrity over extended periods remains a challenge. Our study compared air–liquid interface (ALI) cultivation methods with two different oxygen exposures and evaluated their impact on long-term viability, survival, oxidative stress, and cell integrity.

Submerged cultivation, the conventional method, presents significant limitations, particularly regarding gas exchange and mechanical stress caused by rocking platforms^13^. Our findings confirm that ALI cultivation provides a suitable alternative by allowing direct oxygen exposure and reducing mechanical damage^9^. Moreover, ALI cultivation provides several practical benefits. It streamlines medium changes by allowing the entire insert to be transferred to fresh medium, eliminating the need for direct pipetting into the well. This not only saves time but also minimizes the risk of mechanical damage to cPCTS. Additionally, ALI cultivation enabled long-term microscopy without requiring fixation to the culture plate bottom, as is necessary in submerged conditions.

Oxygen levels play a crucial role in cPCTS integrity and functionality^14^. While high oxygen concentrations (80%) resulted in progressive crypt deterioration over time, reducing oxygen to more physiological levels (atmospheric oxygen) improved the expression of proliferation- and stem cell-associated markers, particularly beyond 72 h. These findings are consistent with previous studies demonstrating that excessive oxygen exposure induces oxidative stress and compromises tissue viability. Although atmospheric oxygen still exceeds the physiological oxygen tensions reported for colonic tissue, it represents a substantial improvement over conventional hyperoxic culture conditions. Lower, more physiologically relevant oxygen levels (approximately 2–9%) would more closely mimic the in vivo environment. Nevertheless, their implementation in cPCTS cultures remains technically challenging. Stable control of reduced oxygen concentrations requires specialized incubation systems. Furthermore, given the lack of vascular perfusion and the reliance on passive diffusion within relatively thick tissue slices, we hypothesize that further reductions in oxygen tension could increase the risk of hypoxia and secondary tissue injury in the central regions of the slices. This hypothesis remains to be tested experimentally. Therefore, atmospheric oxygen may represent a practical compromise between physiological relevance and sufficient oxygen supply to maintain tissue integrity during prolonged ex vivo culture.

We investigated a broader panel of inflammation- and fibrosis-associated markers to gain a comprehensive overview of the condition of the cPCTS. The concomitant induction of *Tgfb1*, *Serpinh1* and *Fn1* gene expression indicates that the model evolves into a reactive, profibrotic stromal microenvironment, particularly at later time points. These findings are likely due to an increased production of fibrous matrix components combined with a simultaneous reduction in matrix degradation by MMPs, ultimately leading to abnormal accumulation of extracellular matrix. Together with lower *Il6* expression, these data suggest more favorable physiological conditions that support mucosal healing following physical surface injury upon atmospheric oxygen^15^.

This process, known as epithelial restitution, is commonly observed during wound healing and is characterized by increased epithelial cell proliferation and differentiation. In cPCTS it is likely, at least in part, a consequence of tissue injury induced during slicing and may therefore reflect a wound healing response rather than steady-state epithelial turnover. Consequently, the observed differentiation and remodeling patterns should be interpreted in the context of injury-driven repair processes. While this limits direct comparison to a “healthy” intestinal epithelium, it also underscores the value of cPCTS as a model to study epithelial repair mechanisms ex vivo. Indeed, we observed a time-dependent increase in proliferating epithelial cells, along with enhanced activation of goblet cells. This coordinated interplay between various mucosal cell types appears to strengthen the intestinal barrier function, which is essential for effective mucosal healing^16^. Overall, the observed inflammatory patterns especially at later time points resemble those typically seen in inflammatory bowel diseases^17^. We are confident that a reduction in oxygen levels diminishes inflammation and is beneficial for the long-term viability of the tissue slice culture. Indeed, the number of Ki67 positive cells was also increased in lung PCTS upon reduced oxygen exposure^14^. Additionally, our data highlight the need for further investigation into the impact of oxygen gradients within the tissue. In the ALI configuration, oxygen supply is not limited to diffusion from the culture medium. The apical surface is directly exposed to the gas phase, allowing oxygen to enter the upper tissue layers, while additional oxygen may be provided from the basal side via the medium. The relative contribution of these two pathways likely depends on factors such as tissue thickness and metabolic activity. The oxygen gradient might also contribute to discrepancies between qPCR data, where RNA is extracted from the entire tissue slice, and histological findings, which are typically assessed from the middle layers^18^. In human body, cells facing the lumen of the colon are exposed to approximately 0.4% oxygen, while those in the muscle layer encounter levels up to 10%^19^. This physiological hypoxia plays a rather complex role in the gut mucosa: hypoxia-induced genes help sustain epithelial barrier integrity, support nutrient absorption, and regulate immune responses, but prolonged HIF activation can worsen disease states, promoting intestinal damage, inflammation, and colorectal cancer^19^. Moreover, physiological hypoxia even impacts the microbiome and susceptibility to antibiotics^20^. However, the precise influence of the microbiome on cPCTS survival requires further investigation.

Despite the benefits of ALI cultivation, our results indicate a gradual decline in crypt integrity after 120 h, with only isolated crypts remaining. This suggests potential limitations in complete crypt regeneration, possibly due to insufficient neuronal regulation, lack of vascular support, or inadequate nutrient availability. Additionally, the emptying of goblet cells during cultivation highlights the need to investigate mucosal stress responses in this model.

## Conclusion

Our study demonstrates that ALI cultivation provides a promising approach for maintaining cPCTS viability at least for 96 h, with reduced oxidative stress, DNA damage, and increased stemness preservation at atmospheric oxygen. This method simplifies medium changes and enables real-time microscopic analysis without additional fixation steps. Optimizing the cutting process by minimizing mechanical stress could reduce fibrotic and inflammatory reactions and improve the tissue integrity.

## Supplementary Information

Supplementary Information.

## Data Availability

Data will be available upon request.
