# Supplementary material for: Optimized air–liquid interface cultivation enhances survival and integrity of mouse colon precision-cut tissue slices
Source: Sci Rep. 2026 Sep 9;16:28238. doi: 10.1038/s41598-026-67282-7 (PMC13558742; doi:10.1038/s41598-026-67282-7)
Supplement: Supplementary file 1 [file 41598_2026_67282_MOESM1_ESM.zip › Suppl. Tables 1-3.docx]

Suppl. Table 1: Compositions of KHB buffer.

| **Reagent** | **1x m [mmol/l]** | **Company** |
| --- | --- | --- |
| CaCl_2_·2H_2_O | 2,48 | Sigma-Aldrich Chemie GmbH, Taufkirchen, Germany |
| KCl | 5,03 | Carl Roth GmbH + Co. KG, Karlsruhe, Germany |
| NaCl | 118,06 | Carl Roth GmbH + Co. KG, Karlsruhe, Germany |
| MgSO_4_·7H_2_O | 1,10 | Sigma-Aldrich Chemie GmbH, Taufkirchen, Germany |
| KH_2_PO_4_ | 1,21 | Carl Roth GmbH + Co. KG, Karlsruhe, Germany |
| NaHCO_3_ | 25,00 | Carl Roth GmbH + Co. KG, Karlsruhe, Germany |
| D-Glucose | 27,53 | Sigma-Aldrich Chemie GmbH, Taufkirchen, Germany |
| HEPES | 10,00 | Sigma-Aldrich Chemie GmbH, Taufkirchen, Germany |

Suppl. Table 2: QPCR primer pairs used in the study.

| **Target** | **Oligonucleotides 5‘-3‘** | **Annealing temperature [°C]** | **Product size [bp]** |
| --- | --- | --- | --- |
| *Alpi* | **Sense:** AGGATCCATCTGTCCTTTGG | 56 | 275 |
|  | **Antisense:** ACGTTGTATGTCTTGGACAG | 55 |  |
| *Atoh1* | **Sense:** ACAGCGATGATGGCACAGAA | 60 | 168 |
|  | **Antisense:** GTTGCCTTCCTAACTGGCCT | 60 |  |
| *Cdh1* | **Sense:** GAGGTCTACACCTTCCCGGT | 61 | 130 |
|  | **Antisense:** CCACTTTGAATCGGGAGTCT | 57 |  |
| *Chga* | **Sense:** CTCGTCCACTCTTTCCGCAC | 61 | 176 |
|  | **Antisense:** CTGGGTTTGGACAGCGAGTC | 61 |  |
| *Cldn15* | **Sense:** GCTTCTTCATGTCAGCCCTG | 58 | 313 |
|  | **Antisense:** TTCTTGGAGAGATCCATGTTGC | 59 |  |
| *Eef2* | **Sense:** GAAGAAGCTGTGGGGAGACC | 60 | 141 |
|  | **Antisense:** ATGGCGTCGAACACCTTGAA | 58 |  |
| *Fn1* | **Sense:** GTTCAAACACCCCTCCCACA | 59 | 270 |
|  | **Antisense:** TGACGTCCACATCATGGAATG | 59 |  |
| *Gclc* | **Sense:** CCTGGAGCCTCTGAAGAACA | 60 | 247 |
|  | **Antisense:** AGACTCGTTGGCATCATCCA | 60 |  |
| *Gclm* | **Sense:** CAGTGGGCACAGGTAAAACC | 60 | 224 |
|  | **Antisense:** GAGTACCTCAGCAGCCACAG | 60 |  |
| *Hmox1* | **Sense:** GCTAGCCTGGTGCAAGATACT | 59 | 120 |
|  | **Antisense:** TGCCAACAGGAAGCTGAGAG | 60 |  |
| *Il1b* | **Sense:** TGCCACCTTTTGACAGTGATG | 60 | 164 |
|  | **Antisense:** TGATACTGCCTGCCTGAAGC | 58 |  |
| *Il6* | **Sense:** GCCTTCTTGGGACTGATGCT | 60 | 181 |
|  | **Antisense:** TGCCATTGCACAACTCTTTTC | 60 |  |
| *Lgr5* | **Sense:** ACCCGCCAGTCTCCTACATC | 61 | 198 |
|  | **Antisense:** GCATCTAGGCGCAGGGATTG | 61 |  |
| *Mki67* | **Sense:** ACCATCATTGACCGCTCCTT | 59 | 150 |
|  | **Antisense:** TTGACCTTCCCCATCAGGGT | 60 |  |
| *Mmp3* | **Sense:** CAGCATCCCCTGATGTCCTC | 60 | 151 |
|  | **Antisense:** TTGCGCCAAAAGTGCCTGT | 61 |  |
| *Msn* | **Sense:** GGAGTCCTTGCCGAAGTGTA | 59 | 116 |
|  | **Antisense:** TGACACGCACACTGATCGTC | 60 |  |
| *Muc2* | **Sense:** ATGCCCACCTCCTCAAAGAC | 60 | 101 |
|  | **Antisense:** GTAGTTTCCGTTGGAACAGTGAA | 59 |  |
| *Nqo1* | **Sense:** GAAGCTGCAGACCTGGTGAT | 60 | 96 |
|  | **Antisense:** TCCTGCTACGAGCACTCTCT | 60 |  |
| *Serpinh1* | **Sense:** CCTGCAGTCCATCAACGAGT | 60 | 132 |
|  | **Antisense:** TTCTCATCCCAGTGTGGCTT | 59 |  |
| *Tgfb1* | **Sense:** GCTGAACCAAGGAGACGGAA | 60 | 138 |
|  | **Antisense:** ATGTCATGGATGGTGCCCAG | 60 |  |
| *Tnfa* | **Sense:** GGACTAGCCAGGAGGGAGAA | 60 | 162 |
|  | **Antisense:** CGCGGATCATGCTTTCTGTG | 60 |  |
| *Vil1* | **Sense:** ATCTCCCTGAGGGTGTGGAC | 61 | 95 |
|  | **Antisense:** AGAGAAGGCAGCTGGAGTCA | 61 |  |

Suppl. Table 3: Antibodies used for immunohistochemestry

| **Primary antibody** | **Cat.-No.** | **Manufacturer** | **Dilution** |
| --- | --- | --- | --- |
| Mouse monoclonal anti-Ki67 (Mib‑1) | M7240 | Dako/Agilent | 1:100 |
| Anti-Villin antibody (SP145) | ab130751 | Abcam | 1:200 |
| Anti-gamma H2A.X (phospho S139) antibody (9F3) | ab26350 | Abcam | 1:2000 |
| Anti-MMP9 antibody | Ab38898 | Abcam | 1:2000 |
| **Secondary antibody** | **Cat.-No.** | **Manufacturer** | **Dilution** |
| Goat polyclonal anti-rabbit (biotinylated) | BA-1000 | Vector Laboratories | 1:100 |
